# Integrated Proteotranscriptomics of Human Myometrium in Labor Landscape Reveals the Increased Molecular Associated With Inflammation Under Hypoxia Stress

**DOI:** 10.3389/fimmu.2021.722816

**Published:** 2021-10-04

**Authors:** Lina Chen, Lele Wang, Yihong Luo, Qian Huang, Kaiyuan Ji, Junjie Bao, Huishu Liu

**Affiliations:** ^1^ School of Medicine, South China University of Technology, Guangzhou, China; ^2^ Guangzhou Key Laboratory of Maternal-Fetal Medicine, Guangzhou Women and Children’s Medical Center, Guangzhou Medical University, Guangzhou, China

**Keywords:** labor, myometrium, transcriptome, proteome, inflammation

## Abstract

During labor, a variety of coordinated physiological and biochemical events cause the myometrium to transition from a quiescent to contractile state; the molecular mechanisms responsible for this transition, however, remain unclear. To better understand this transition at a molecular level, the global transcriptome and proteome of human myometrial samples in labor and those not in labor were investigated through RNA sequencing (RNA-seq) and quantitative liquid chromatography–tandem mass spectrometry (LC-MS/MS) *via* data-independent acquisition (DIA) and parallel reaction monitoring (PRM) methods. Furthermore, an integrated proteotranscriptomic analysis was performed to explore biological processes and pathway alterations during labor; this analysis identified 1,626 differentially expressed mRNAs (1,101 upregulated, 525 downregulated) and 135 differentially expressed proteins (97 upregulated, 38 downregulated) in myometrium between nonlabor and in labor groups. The comprehensive results of these analyses showed that the upregulated mRNAs and proteins increased inflammation under hypoxia stress in the myometrium under labor, and related proteins and cytokines were validated by PRM and Luminex assays. Our study confirmed the biological process of inflammation and hypoxia in laboring myometrium at the transcriptome and proteome levels and provided recourse to discover new molecular and biological changes during labor.

## Introduction

During human pregnancy, the uterus, especially the myometrium, remains quiescence. However, during labor, the myometrium undergoes biochemical and structural changes, transforming from a relaxed, noncontractile phenotype to a highly coordinated and intensely contractile one. The most accepted theory is the interaction between steroid hormones, paracrine molecules, and inflammation during contraction with rhythmic hypoxia ischemia (1, 2); these factors seem to play an important role, yet the exact mechanism remains ambiguous.

The transition of a quiescent myometrium state to an active contractive one requires complex and highly regulated changes in gene coding to drive changes in the structure, contractility, and signaling in the uterine myometrium (3, 4). RNA-seq has now become the most commonly used method for transcriptome analysis; the complete sequence of mRNA in human myometrium, whether in quiescent or active states, has been sequenced and recorded by RNA-seq in previous studies (5). Stanfield et al. integrated three transcriptome datasets in the NCBI Gene Expression Omnibus database to characterize 126 labor-associated genes; labor signatures include interleukins, cytokines, apoptosis, MYC, and cell proliferation/differentiation, whereas cyclic AMP signal and muscle relaxation pathway were mainly associated with nonlabor (6). These signatures accurately categorize and describe the various stages of delivery. However, despite the generation of some muscular transcriptome datasets, reliable human delivery transcriptional signals and pathway networks have yet to be studied; moreover, extensive and consistent studies and review comparisons are lacking.

It is quite challenging to elucidate the mechanism of labor based on pure transcriptome data without information on the changes in the proteome, as proteins are responsible for carrying out biological functions. Prior studies have attempted to detect human myometrium proteome using two-dimensional gel electrophoresis (2-DE) and mass spectrometry (7) or two-dimensional liquid chromatography coupled with tandem mass spectrometry (8). However, these have not been very successful as the accuracy and sensitivity of protein detection have been limited, resulting in many protein signals lost. In recent years, the development of mass spectrometry technology, either untargeted or targeted approaches, had fortunately made it possible for high throughput protein qualitative and quantitative detection (9). Although proteomics was extensively utilized in disease research, its application in studying delivery mechanism has not been reported to date.

Further systematic landscape depicting the relationship between the proteome and transcriptome in the myometrium during labor, beyond a single technique, is a more powerful technique to explore the molecular mechanisms involved in labor.

Based on these considerations, RNA-seq and LC-MS/MS DIA coupled with PRM technology were employed to identify globally mRNAs and protein abundance changes in myometrial tissues collected from women nonlabor and in labor. Furthermore, integrated analysis between transcriptome and proteome, recognized as proteotranscriptomic analysis, was performed to reveal core genes, biological processes, and signaling pathways in labor.

## Materials and Methods

### Subjects and Tissue Collection

Myometrial tissue samples were collected from lower segment of 40 women with singleton gestations undergoing cesarean deliveries at Guangzhou Women and Children’s Medical Center (Guangzhou, China). These patients were undergoing caesarean section for reasons of breech or large baby/cephalopelvic disproportion, with no pregnancy complications (such as gestational diabetes, preeclampsia, and chorioamnionitis), or given labor-augmenting drugs (prostaglandins and oxytocin). Subjects were recruited into two groups: spontaneous term in labor (IL, n = 20) and nonlabor (NL, n = 20). Labor was defined as regular contractions (<3 min apart) and cervical dilatation (>2 cm). Tissues were stored at -80°C. Clinical and pathological information was obtained from medical records and pathology reports. All research was approved by the ethics committee of Guangzhou Women and Children Medical Center (No. 201915401) for the protection of human subjects.

### mRNA Sequencing

Total RNA of myometrium samples was extracted from the tissues using Trizol (Invitrogen, Carlsbad, CA, USA) according to the manual instructions (10). The tissue samples were ground to approximately 60 mg powder using liquid nitrogen and transferred to a tube containing 1.5 ml of Trizol reagent. The mixture was centrifuged at 12,000×g for 5 min at 4°C. The supernatant was transferred to a new tube to which 0.3 ml of chloroform/isoamyl alcohol (24:1) per 1.5 ml of Trizol reagent was added. After the mixture was centrifuged at 12,000×g for 10 min at 4°C, the aqueous phase was transferred to a new tube that contained an equal volume of the supernatant of isopropyl alcohol. The mixture was centrifuged at 12,000×g for 20 min at 4°C, and then the supernatant was removed. After being washed by 75% ethanol, the RNA pellet was air-dried in the biosafety cabinet and then dissolved by DEPC-treated water. Total RNA was qualified and quantified using a Nano Drop and Agilent 2100 bioanalyzer (Thermo Fisher Scientific, MA, USA).

Both cytoplasmic and mitochondrial ribosome RNAs were removed from total RNA preparations using target-specific oligos and RNase H reagents. Following SPRI bead purification, RNA was fragmented into small pieces using divalent cations at elevated temperature. The cleaved RNA fragments were copied into the first strand cDNA using reverse transcriptase and random primers, and a second strand cDNA was synthesized using DNA Polymerase I and RNase H. This process removed the RNA template and synthesizes a replacement strand, and ds cDNA was synthesized using dUTP instead of dTTP. These cDNA fragments were then added with an “A” base and subsequently ligated to an adapter. After treatment with uracil-DNA glycocasylase, the second chain was quenched by the addition of dUTP during amplification. The products were enriched with PCR to create the final cDNA library. The libraries’ quality and quantity were assessed in two methods: checking the distribution of the fragment size using the Agilent 2100 bioanalyzer, and quantifying the library using real-time quantitative PCR (qPCR) (TaqMan Probe). The qualified libraries were sequenced pair end on the BGISEQ-500/MGISEQ-2000 System (BGI-Shenzhen, China).

The mRNA quantitative expression values were calculated for each sample based on the number of fragments per kilobase of exon per million fragments mapped (FPKM). The process of gene mapping alignment uses HISAT2 based on the *Homo sapiens* UCSC hg38 reference (GRCh38) genome (https://ccb.jhu.edu/software/hisat2/index.shtml). Alignment calculations of mRNA are performed using StringTie based on the Ensembl genome browser 84 in a GTF file format (ftp://ftp.ensembl.org/pub/release-84/gtf/homo_sapiens) (11). The data presented in the study are deposited in the GEO repository, accession number GSE181348.

### Protein Extraction and Peptide Purification

All of the frozen myometrium samples were ground and homogenized in RIPA lysis buffer and protease inhibitor cocktail tablets (PhosSTOP, Roche), and subsequently homogenized by sonication (Scientz) on ice. The homogenate was centrifuged at 12,000g for 15 min at 4°C. Supernatants were transferred into clean tubes. Protein samples were digested and referred to filter-aided sample preparation (FASP) (12). The protein solutions were diluted with Buffer 1 (8M urea/100 mM Tris-HCl, pH 8.5) and were centrifuged. Proteins were reduced with 100 mM DL-Dithiothreitol for 2 h at 37°C. Subsequently, the samples were alkylated in the dark using 50 mM iodoacetamide for 30 min. The protein solutions were centrifuged and washed twice by Buffer 2 (8M urea/100 mM Tris-HCl, pH 8.0), and the concentrate was subjected to proteolytic digestion overnight at 37°C. The digests were collected by centrifugation. The purity and quantity of protein and peptides were determined by an automatic analyzer (Hiachi7600-020, Japan).

### LC-MS/MS Analysis

The peptides of myometrium samples were separated on a Thermo Ultimate 3000 UHPLC analytical column (ZORBAX Extended-C18, 2.1, Agilent). Mobile phases A (10 mM ammonium formate, 5% acetonitrile, pH 10.0) and B (10 mM ammonium formate, 90% acetonitrile, pH 10.0, 80 min gradient from 5% to 38%) were used to develop a gradient elution, at 0.3 ml/min flow rate, and were monitored at UV 214 nm. Sixteen fractions were collected a tube per minute and vacuum-dried for subsequent data-dependent acquisition (DDA) analysis and DIA analysis.

DDA analysis was performed using Easy nLC 1200 nanoflow liquid chromatography system (Thermofisher, USA) and Thermofisher Q Exactive system coupled with Nanospray Flex Ion Source (Thermofisher, USA) operating in the DDA mode. The peptide digests were reconstituted in Nano-LC mobile phase A (0.1% formic acid, FA) and loaded onto a NanoViper C18 trap column (3 μm, 100 Å), then separated onto an analytical column (75 μm×25 cm C18-2 μm 100 Å), using a 120-min gradient from 5% to 38% mobile of phase B (80% acetonitrile, 0.1% FA). The Q Exactive mass spectrometer was operated with a spray voltage of 1.9 kV and a capillary temperature of 275°C, in 350–2,000 m/z scan range and 70,000 resolution; the maximum ion injection time was 100 ms. Twenty most abundant precursor ions from each DDA cycle were selected for higher energy collision dissociation (HCD) fragment analysis with a maximum ion injection time of 50 ms, high-energy collision energy of 28 eV, and a dynamic exclusion of 25 s.

For each sample DIA analysis, MS1 and MS2 were set with a maximum ion injection time of 50 ms. MS1 was set with a resolution of 70,000, 350–1,200 m/z scan ranging, which was separated into 34 acquisition windows with a mass range of 14–152 Da. The liquid conditions were consistent with the DDA model for separation. DIA raw data were analyzed by Spectre Pulsar X.

### Spectral Library Construction

The spectral library of the myometrium samples was built up by raw MS files and searched against the Human UniProt protein database (20,336 protein sequence) analyzed by Proteome Discoverer (V2.1.0.81). The search was conducted with a precursor tolerance of 10 ppm and a fragment ion tolerance of 0.05 Da. Identified peptides were filtered with false discovery rate (FDR) < 5% and containing at least one Unique peptide segment. Protein and peptide FDR < 1%. The protein abundance was calculated by the peak areas of their samples. The data presented in the study are deposited in the ProteomeXchange repository, accession number PXD027906.

### Data Bioinformatics Analysis

Differential expressed (DE) mRNA and protein in IL *vs* NL were identified using two-sided Student’s t-test through OmicShare tools (http://www.omicshare.com/tools), based on two criteria of 1.5-fold change (FC) increase or decrease in expression levels and p-value < 0.05. Gene ontology (GO) terms in biological processes (BP), cellular components (CC), and molecular functions (MF) were enriched using DAVID v6.8, taking p-value < 0.05 as a threshold. Pathway analysis was conducted using KOBAS 3.0, and a p-value < 0.05, corrected by Benjamini-Hochberg method, was considered significant (13, 14). Principal component analysis (PCA) was conducted using the PCA function in the R package FactoMineR. PANTHER was used for protein classification (15). Gene set enrichment analysis (GSEA) was performed using the pre-ranked method in GSEA Java implementation (16), Molecular signatures database (MsigDB, http://software.broadinstitute.org/gsea/msigdb) was used for the gene sets (17); for each category, a p-value < 0.05 was considered significant. The protein–protein interaction (PPI) network of DE proteins was constructed by the Cytoscape (version 3.7.2) STRING APP tool (18). Correlation of mRNAs and protein expression levels was generated using the R corrplot function.

### PRM Analysis

LC-MS/MS methods were the same as DIA-MS. The peptide digests were separate and analyzed by Easy nLC and Q-Exactive mass spectrometry. The target protein data were imported into the Skyline software and inserted into the iRT peptide (TPVITGAPYEYR). The data were imported into the original data of DDA to screen the matching peptides with high ionic strength and fewer heteropeaks. The ion flow information, peak area, and elution time of the peptide segment were obtained. Samples were analyzed by full scan followed by the PRM mode. The Skyline software selected peptides based on mass error less than 10 ppm, examined peak area, and elution time. The peak area was corrected with internal standard peptide (19).

### Luminex Assay

The protein extraction procedure is the same as the one described above. Take 50 ul of each sample for multiplex assay with a Bio-Plex Pro Mouse Cytokine 7-Plex Array kit (Bio-Rad). Proteins were incubated with antibodies conjugated to Magplex microspheres under shaking for 2 h at room temperature. Then they were incubated with antibodies for 1 h and streptavidin phycoerythrin for 30 min at room temperature. Finally, the protein concentration was detected by Luminex 200 system and Bioplex HTF (Bio-Rad) and calculated according to standard sample protein concentration.

### Statistical Analysis

Group comparisons were performed using the t test for two groups and ANOVA test for multiple groups by SPSS 20.0; data were expressed as means ± standard error, and p value ≤ 0.05 was considered significant.

## Results

### Clinical Characteristics of Myometrial Samples

**Figure 1:**
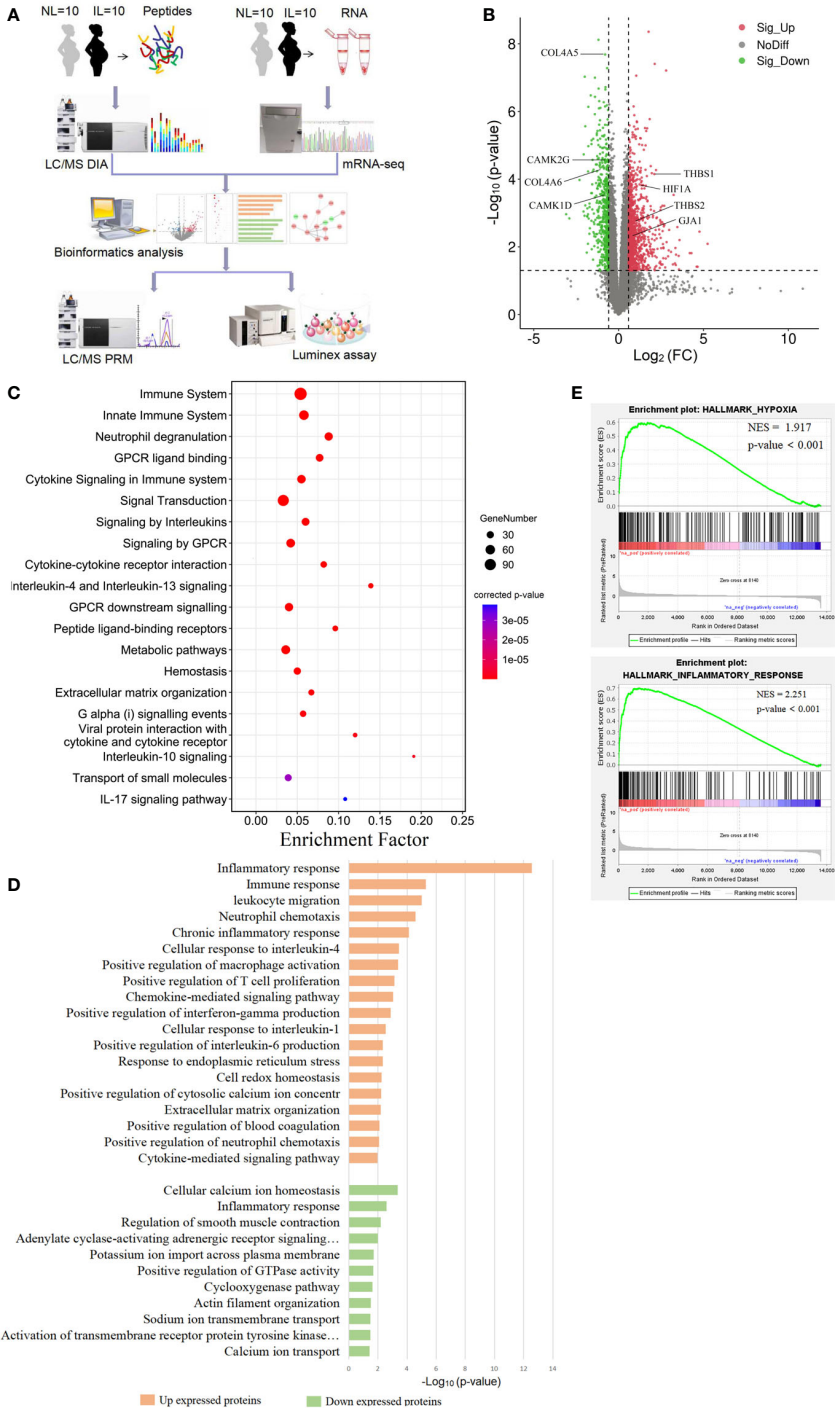
Visualization of the transcriptomics results of mRNAs between nonlabor and in labor myometrium. **(A)** Overall study design and workflow. **(B)** Volcano plot of all mRNA alteration between NL and IL groups. The horizontal dashed line corresponds to p value < 0.05, and the vertical dashed line corresponds to a 1.5-fold change decrease or increase in expression levels. Red, green, and black dots represent upregulated, downregulated, and nondifferentially expressed transcripts in the IL group (as compared to the NL group), respectively. Data associated with this figure can be found in **Supplemental Table 1**. **(C)** Bubble diagram of partial significantly enriched pathways for DE mRNAs. **(D)** Bar diagram of partial biology progress GO term clusters of DE mRNAs. **(E)** GSEA plots of mRNA sets of hypoxia and inflammatory response.

The clinical details of patients from whom myometrial tissues (n=40) were collected are provided in **Table 1**. The mRNA transcripts in 20 samples (IL, n=10; NL, n=10) were quantified using mRNA-seq. Additional 20 samples (IL, n=10; NL, n=10) were investigated using LC-MS/MS DIA to identify and quantify protein, and further used PRM to validate the DIA results (**Figure 1A**). Results showed no statistically significant differences in maternal age, parity, BMI, and gestational age (GA) at delivery among the four groups (p>0.05).

**Table 1:** Characteristics of the women who provided myometrium samples.

	Maternal age (years)	Parity	BMI (kg/m^2^)	GA at delivery (weeks)
mRNA sequencing				
NL (n=10)	29.20 ± 4.78	1.40 ± 0.70	24.93 ± 1.92	39.04 ± 0.51
IL (n=10)	29.10 ± 2.60	1.50 ± 0.97	25.93 ± 2.04	39.36 ± 0.71
LC-MS/MS				
NL (n=10)	32.00 ± 4.78	1.60 ± 0.84	25.96 ± 4.85	38.81 ± 0.78
IL (n=10)	28.65 ± 2.97	1.20 ± 0.42	27.15 ± 3.40	39.12 ± 1.04
p value	0.248	0.683	0.559	0.474

Data are expressed as mean ± SEM. NL, nonlabor; IL, in labor; BMI, body mass index; GA, gestational age; LC-MS/MS, liquid chromatography–tandem mass spectrometry.

### DE mRNA in Quiescent and Laboring Myometrium

To establish a thorough transcriptome profile of NL and IL myometrium, we conducted an investigation through RNA-seq on mRNA separated from the myometrium of 10 NL and 10 IL women. A total of 13,602 distinct mRNAs were detected after excluding mRNAs that were present at extremely low abundances (average FPKM<1 in both NL and IL). A total of 1,626 DE transcripts were identified; of these, 1,101 were upregulated and 525 were downregulated transcripts in active labor compared to those with quiescent myometrium. Among the upregulated mRNA, there were several known prominent labor-associated players including (but not limited to) gap junction alpha-1 protein (GJA1/CX43) and adhesion molecules (vascular adhesion molecule 1, VCAM1; thrombospondin 1, THBS1, 2 THBS2; carcinoembryonic antigen-related cell adhesion molecule 1, CEACAM1). Hypoxia-inducible factor 1 alpha (HIF1A), a remarkable gene regulating the hypoxia stress response, was significantly upregulated in IL compared to that of NL. In addition, a number of genes known to be associated with uterine quiescence appeared in the downregulated transcript set, including (but not limited to) mRNAs encoding proteins associated to cell extracellular matrix interactions (Collagens type IV alpha 5, COL4A5, and 6 COL4A6) and proteins involved in regulating calcium signaling (calcium/calmodulin dependent protein kinase ID, CAMK1D, and calcium/calmodulin dependent protein kinase II gamma, CAMK2G) (**Figure 1B** and **Supplemental Table 1**). Such findings demonstrate that substantial transcriptional changes occur in myometrium during labor.

### Functional Enrichment Analysis of DE mRNAs

In order to get a better understanding of the DE mRNA biological effects in labor, KOBAS and DAVID tools were employed to analyze pathway enrichment and determine the GO biological processes, respectively. DE mRNAs significantly enriched 152 pathways, commonly related to immune, metabolic, and hemostasis. The 305 enriched biological processes were involved in inflammation, gene expression, cell growth, cell communication, ion transport, muscle contraction, and other functions. These results showed that various biological changes have taken place in the myometrium during labor. All DE mRNA bioinformatics results statistically significant pathways, BP, CC, and MF were presented in supplemental materials **(** **Supplemental Tables 2, 3** **)**.

According to the results of functional enrichment, inflammation-related pathways and biological processes were the ones that most prominently enriched and the most frequently occurred. There were multiple enriched terms related to interleukin, including interleukin-4, 13, 10, and 17 signaling pathways, and interleukin-1, 4, and 6 response biological processes (**Figures 1C, D** **)**. The immune response was another significant cluster in upregulated mRNA that involves a variety of immune cell positive regulations, such as macrophage, T cell, and neutrophil. These biological processes combine multiple chemokines and interleukin containing CC chemokine subfamily (CCL13, CCL21, CCL2, CCL23 and CCL11), CXC chemokine subfamily (CXCL1, CXCL8 and CXCL6), and interleukin (IL15, IL2, IL24, and IL10) (**Figure 1D**). Given that delivery was in hypoxic environments (20), we noticed that the HIF-1 signaling pathway was expectedly enriched. We then performed GSEA to gain a broader understanding of the changes in the myometrium during labor. The results showed that hypoxia and inflammatory responses were positively enriched (**Figure 1E**).

Other biological processes were also important. Metabolism of substances such as proteins, lipids, and nucleotides showed highly significant enrichment. Combined with the biological oxidation plasma pathway, it seems that the energy supply had a significant effect on laboring (**Figure 1C**). The biological processes linked with downregulated transcripts in IL *vs* NL were mainly clustered in ion transport, especially the regulation of calcium ions, which may be closely related to muscle contraction regulation (**Figure 1D**).

### Differential Protein Expression in Quiescent and Laboring Myometrium

**Figure 2:**
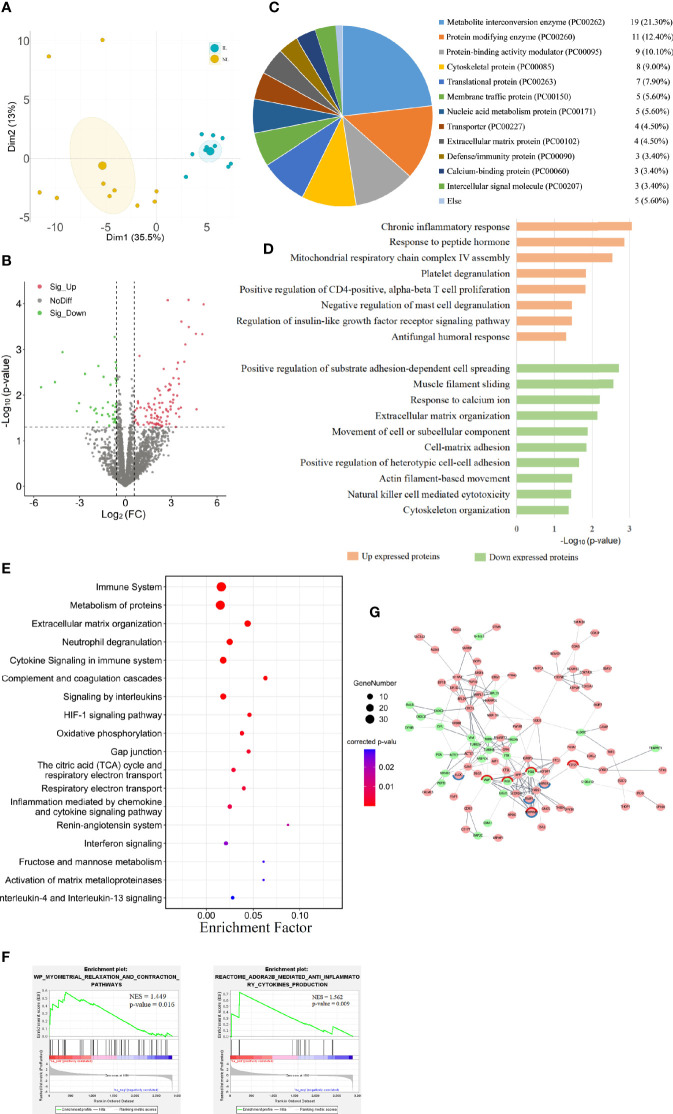
Visualization of the proteomic results between nonlabor and in labor myometrium. **(A)** PCA plot based on DE proteins. IL samples (blue dots) clustered distinctly from NL (yellow dots). **(B)** Volcano plot of all protein alteration between NL and IL groups. The horizontal dashed line corresponds to p value < 0.05, and the vertical dashed line corresponds to a 1.5-fold change decrease or increase. Red, green, and black dots represent upregulated, downregulated, and nondifferentially expressed transcripts in the IL group (as compared to the NL group), respectively. Data associated with this figure can be found in **Supplemental Table 4**. **(C)** Pie graph for classification of DE proteins showing the overall percentages of types to the DE proteins. **(D)** Bar diagram of partial biology progress GO term clusters of DE proteins. **(E)** Bubble diagram of partial significantly enriched pathways for DE proteins. **(F)** GSEA plots of interest sets of myometrial relaxation and contraction pathways and ADORA2B mediated anti-inflammatory cytokine production. **(G)** Protein–protein interactions between DE proteins. Pink circles represent upregulated proteins, green circles represent downregulated proteins, and the circles with red edges and blue edges represent complement and coagulation cascades and HIF-1 signaling pathway key proteins, respectively.

To discover the changes in protein expression, we performed DIA proteomic analysis to quantify protein abundance. A total of 2,868 proteins were quantified in 10 IL and 10 NL myometrium. PCA of the DE proteins showed that the protein expression profile of the IL group was clearly separated from that of the NL group (**Figure 2A**), suggesting some unique confounding proteomic features for the IL cases. We found that 135 DE proteins contain 97 upregulated proteins and 38 downregulated proteins in IL *vs* NL. Statistical results of protein quantification are shown in a volcano plot (**Figure 2B** and **Supplemental Table 4**). Protein classification indicated that 89 of the DE proteins were grouped into 17 classes, with the basic functions of these falling under catalysis, transport, binding, signal transduction, immune, and cell connectivity. Among them, metabolite interconversion enzyme and protein modifying enzyme accounted for a large proportion, consisting of 19 and 11 proteins, respectively (**Figure 2C**).

### Functional Enrichment Analysis of DE Proteins

The GO analysis showed that 21 biological processes were enriched in the upregulated proteins in IL *vs* NL, commonly related to inflammation and immune responses, cell proliferation, humoral response, and gene expression. The downregulated proteins in IL *vs* NL clustered 24 distinct biological processes, mainly involved in cytoskeleton, matrix organization, peptide hormone secretion regulation, blood coagulation, and ion response. The pathway enrichment analysis enriched 158 pathways, commonly related to immune system, cell junction, blood coagulation, cell movement, and other pathways. All DE protein bioinformatics results, statistically significant pathways, BP, CC, and MF are presented in supplemental materials (**Supplemental Tables 5, 6**).

The results of functional analysis of DE protein were partially similar to those of DE mRNAs. The proteins upregulated in the IL group were mostly significantly enriched in inflammation and immune responses, including chronic inflammatory responses, T cell, and mast cell regulation biological processes (**Figure 2D**). Immune system was the most enriched pathway. Moreover, some immune-related pathways, such as neutrophil degranulation, cytokine signaling in immune system, and complement and coagulation cascades were highly enriched. Interleukins and the HIF-1 signaling pathway were also significantly enriched (**Figure 2E**).

To further capture the functional enrichment, we accomplished a GSEA. The results implicated that two positively enriched protein sets were those of myometrial relaxation and contraction pathways, as well as ADORA2B mediated anti-inflammatory cytokine production (**Figure 2F**). By constructing a network of interactions between the DE proteins, we can further discover the correlation between the functional proteins. The key proteins of complement and coagulation cascades (VWF, FGG, SERPINE1, FGB, and CD55) and the HIF-1 signaling pathway (ELOC, HMOX1, TIMP1, and SERPINE1) form the tightest cluster of proteins in the protein interaction diagram (**Figure 2G**).

### Integration of the Transcriptomics and Proteomics

**Figure 3:**
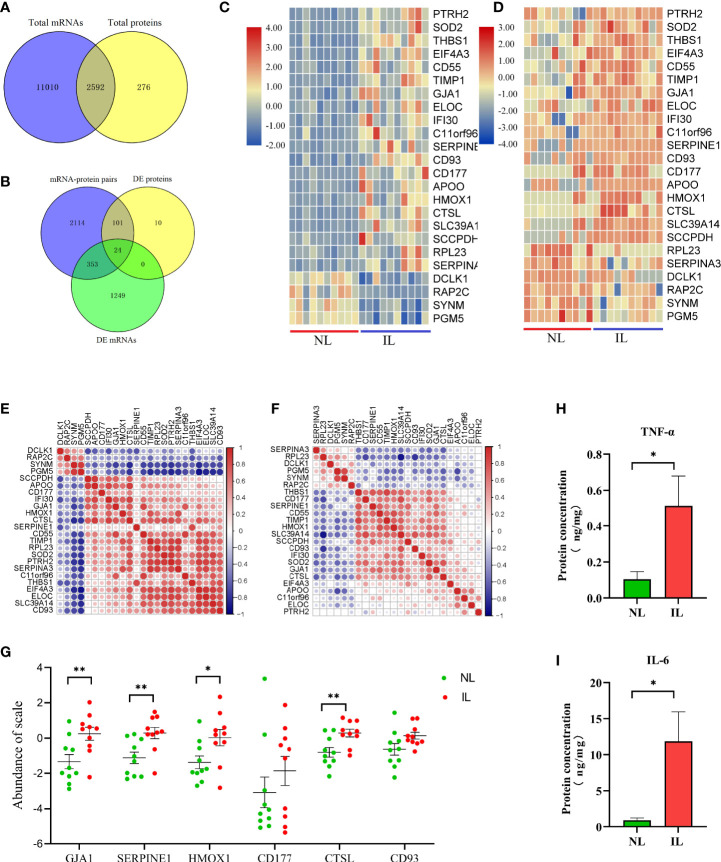
Visualization of the proteotranscriptomic results between nonlabor and in labor myometrium. **(A)** Venn diagram of the gene set identified both in transcriptome and proteome. **(B)** Venn diagram of gene sets of mRNA–protein pairs, DE mRNAs, and DE proteins. **(C, D)** Heatmaps of expression of the 24 overlapping mRNAs **(C)** and proteins **(D)**. The color gradient represents gene expression levels as z-scores. **(E, F)** Correlation matrix of the expression levels of the 24 overlapping mRNAs **(E)** and proteins **(F)**. The darker the dot, the stronger the correlation. Red indicates positive correlation; blue indicates negative correlation. **(G)** Quantification validation of proteins by PRM. **(H, I)** Quantification validation of TNF-α and IL-6 by Luminex assays. *p<0.05, **p<0.01.

Due to the different detection techniques of mRNA and protein detecting, the number of mRNAs and proteins varies greatly. In addition, the mRNA translation into protein is quite complex due to the difference in stability and degradation between the mRNA and the protein. This is why protein expression levels are not always consistent with mRNA. The overlapping of the identified mRNAs and proteins showed 2,952 mRNA–protein pairs coexpressed in the myometrium (**Figure 3A**); of these, 353 only differed in the mRNA level between the IL and NL myometrium, and 101 were post-transcriptionally altered without any accompanying changes in mRNA transcript levels. A total of 24 mRNA–protein pairs were found to be different between the IL and NL myometrium at both mRNA and protein levels (**Figure 3B**). Most of the expression of mRNA–protein pairs presented the same trend of either being upregulated or downregulated, although in two cases (RPL23 and SERPINA3), protein levels were decreased while mRNA levels increased in the IL myometrium. The heat map displayed more obvious mRNA expression; nevertheless, the expression consistency was higher than proteins (**Figures 3C, D** **)**. Correlation analysis of the expression profiles of the 24 mRNA–protein pairs indicated that some of these pairs show highly correlated expression patterns. For instance, SYNM and PGM5 presented a positive correlation both in mRNA and protein (**Figures 3E, F** **)**.

The pathway enrichment analysis of these 24 mRNA–protein pairs showed that the most evident pathway was the immune system; there are also several immune-related clusters such as the innate immune system and processing and presentation of antigen. The clusters of neutrophil degranulation and interleukin-4 and interleukin-13 signaling both belong to the inflammatory response. A variety of proteins were intimately involved in these pathways (CTSL, TIMP1, CD55, HMOX1, CD177, IFI30, and CD93), which was promising for abnormal labor management. Additionally, the HIF-1 signaling pathway was still drastically enriched, and the four genes related to it (ELOC, HMOX1, TIMP1, and SERPINE1) were significantly upregulated at both mRNA and protein levels. The HIF-1 signaling pathway may be the targets of resisting hypoxic pressure during labor to regulate uterine contraction. In addition, related pathways such as coagulation, extracellular matrix, and protein metabolism should not be overlooked (**Table 2**).

**Table 2:** KOBAS pathway enrichment results.

Term	ID	Corrected P-Value	Gene
Immune system	R-HSA-168256	1.278E-06	RAP2C, SERPINA3, CTSL, TIMP1, CD55, HMOX1, CD177, IFI30, CD93, ELOC, EIF4A3
Response to elevated platelet cytosolic Ca^2+^	R-HSA-76005	1.278E-06	SERPINE1, SERPINA3, SCCPDH, TIMP1, THBS1
Platelet activation, signaling, and aggregation	R-HSA-76002	2.463E-05	SERPINE1, SERPINA3, SCCPDH, TIMP1, THBS1
HIF-1 signaling pathway	hsa04066	2.524E-05	HMOX1, ELOC, TIMP1, SERPINE1
Hemostasis	R-HSA-109582	5.230E-05	SERPINA3, TIMP1, CD177, SCCPDH, THBS1, SERPINE1
Neutrophil degranulation	R-HSA-6798695	2.637E-04	RAP2C, SERPINA3, CD55, CD177, CD93
Innate immune system	R-HSA-168249	0.001	RAP2C, SERPINA3, CTSL, CD55, CD177, CD93
Extracellular matrix organization	R-HSA-1474244	0.001	CTSL, SERPINE1, TIMP1, THBS1
Antigen processing and presentation	hsa04612	0.013	CTSL, IFI30
Metabolism of proteins	R-HSA-392499	0.013	PTRH2, TIMP1, CD55, RPL23, THBS1, ELOC
Complement and coagulation cascades	hsa04610	0.013	SERPINE1, CD55
Cytokine signaling in immune system	R-HSA-1280215	0.016	TIMP1, HMOX1, EIF4A3, IFI30
Interleukin-4 and Interleukin-13 signaling	R-HSA-6785807	0.020	HMOX1, TIMP1
Degradation of the extracellular matrix	R-HSA-1474228	0.028	CTSL, TIMP1

### Proteins Validated by PRM

According to the above explained transcriptome and proteomics analysis, whether alone or a combination of both, we found that a large number of molecules associated with immune and inflammation were upregulated in the myometrium during delivery. Furthermore, there was an essential conservative regulation as a response to low oxygen tension and oxidative stress. To further validate these results, some selected candidates who were indicated with important functions, including CTSL, HMOX1, CD177, CD93, and SERPINE1; the expression level of these proteins was validated by PRM. Moreover, the widely confirmed GJA1 protein associated with uterine contraction was also validated. After normalizing the raw protein expression datasets, we found that except for CD177 and CD93, all other proteins showed increased expression as expected; these results were consistent with the DIA results, indicating that the DIA-derived proteomics results for this study were reliable and reproducible (**Figure 3G**).

### Pro-Inflammatory Cytokines in Myometrium

To further verify that the IL myometrium has heightened inflammatory responses, the abundances of two typical inflammation-induced pro-inflammatory cytokines, TNF-α and IL-6, were measured in the NL and IL myometrium. The results revealed that both TNF-α and IL-6 protein concentrations were higher in the IL group, which indicates that, as expected, a heightened inflammatory response is involved in labor (**Figures 3H, I** **)**.

## Discussion

Herein, transcriptome and proteome data in the myometrium of NL and IL were presented; we quantified 13,602 mRNAs and 2,868 proteins in total. The study showed that during labor, mRNA expression levels changed more generally than in corresponding proteins; proteomics describe only a part of the changes detected by the transcriptome. The DE mRNAs and proteins were involved in numerous biological functions of the myometrium, mainly including the regulation of immune response, hypoxic stress, cytoskeleton, cell growth, cell movement, coagulation, and so on. Notably, we found that both mRNAs and proteins upregulated in the IL myometrium were specifically associated with hypoxic stress and inflammation. These results suggest that in time of labor, the human body has a strong protective molecular mechanism against hypoxia and the activation of inflammatory-related responses.

During labor, the strong periodic contractions of the uterine muscles compress muscular vessels, and a marked drop in blood flow can be noticed. It follows that locally transient hypoxia and ischemia are normal phenomena during delivery (20, 21). Surprisingly, however, as uterine muscle contractions become progressively stronger and more frequent as labor progresses, such changes have also been reported in a previous study, where we monitored uterine and abdominal muscle electromyographic activity in nulliparous women during labor (22). Mohammed Alotaibi et al. (23) proposed a novel mechanism, the hypoxia-induced force increase, and this hypoxia response resulted in a pregnancy-specific continuous increase in muscular contractions. The HIF-1 signaling pathway is a key regulatory pathway in hypoxic and ischemic responses (24), which were markedly enriched in our results. The four genes associated with this pathway (ELOC, HMOX1, TIMP1, and SERPINE1) were found to be strongly upregulated in the IL myometrium at both mRNA and protein levels, which may be used to a key regulator in uterine contraction.

Hypoxic stress activates multiple transcription factors that regulate gene expression. Of these, the hypoxia inducible factor 1α (HIF-1α) is the most ubiquitously expressed and considered to be the primary regulator of hypoxia. HIF-1α, which controls oxygen delivery and utilization by regulating angiogenesis and metabolism, plays a critical role in protecting combat ischemic injury (25) and in shaping the hypoxic microenvironments in tumors (26). Our unpublished data have shown that HIF-1α is upregulated in the IL myometrium. The HIF-1 signaling pathway could highly be a vital protective factor for maintaining the uterus contracting vigorously. Some positive biological effects of HMOX1, as an antioxidant, and cytoprotective functions have received considerable attention (27). Studies have shown that the HMOX1 expression is increased in the IL myometrium and exerted pro-inflammatory effects (28). As of now, little is known about the functions of the other proteins involved in the HIF-1 signaling pathway, namely, ELOC, TIMP1, and SERPINE1, in the human myometrium, which requires further research.

During hypoxic stress, many cellular processes change. Biological function analysis of the genes upregulated during labor revealed that the most significant changes occurred in the inflammatory response. A large number of chemokines and cytokines were highly expressed in the IL myometrium; the immune cells (macrophage, T cell, and neutrophil) were activated as well. Many studies have established that hypoxia induces inflammation (29, 30) and that it regulates transcription factors in immune cells including HIF and NF-κB, both of which contribute to macrophage polarization, neutrophil survival, and interferon synthesis; however, these factors also restrict NK cytotoxicity in innate immunity. Considering the adaptive immune cell activity, hypoxia mainly anti-T cell inflammation and pro-B cell survival, and immune cells releasing cytokines, chemokines, and matrix metalloproteinase can further promote inflammation (31, 32).

Inflammation plays an important role in labor (33). Studies in humans and mice have shown that during labor, immune cells such as leukocytes and neutrophils flow into the myometrium and the cervix; the increase in pro-inflammatory cytokines, chemokines, and cell adhesion molecules further amplify inflammation in the labor process (34–36). In our transcriptomic analysis, the neutrophil chemokines CXCL1 and CXCL8 (30) and the monocyte/macrophage chemokine CCL2 (37) were notably upregulated in the IL myometrium, signifying that neutrophils and macrophage are more abundant during labor. The augmentation in multiple CC chemokines, CXC chemokines, and interleukin in the IL myometrium indicated the formation of a proinflammatory microenvironment. Proinflammatory cytokines TNF-α and IL-6 were upregulated in parturient gestational tissue, and their roles in inducing premature delivery have been established (35). In our study, the TNF-α and IL-6 concentration detection also confirmed their up-expression during labor. Combining the results of the biological process and pathway analysis further suggests that there is a global increase in inflammatory processes during labor, and that this response is further regulated by the expression of specific genes at transcription and protein levels.

In addition to the hypoxic and inflammatory responses, our data also indicated that the upregulated genes radically affected the metabolism, which may be due to cellular responses to hypoxic stress, as labor is an energy-intensive process; an active metabolism can ensure adequate supply of energy. Our previous myometrial metabolomic profile analysis presented an increase in metabolism during delivery, especially in lipolysis and fatty acid oxidation (38). Other important pathways enriched in the IL myometrium included those involved in hemostasis and extracellular matrix organization. Procoagulant factor-dependent activation increases immediately after placenta delivery to prevent excessive blood loss, while excessive coagulation increases the risk of venous thromboembolism in puerperal women (39). The results of our analysis indicate that the activity regulation of the coagulation system starts as early as labor. Genes closely related to coagulation function such as SERPINE1 and SERPINA3 may become new coagulation indicators. At parturition, the myometrial extracellular matrix reorganizes to allow contraction and undergoes postpartum involution to revert back to its prepregnancy state. Matrix metalloproteinases (MMPs) are regarded as important regulators of degradation and remodeling of the extracellular matrix (40). In our study, multiple MMP expressions were increased in labor as well as CTSL and THBS1; these may be new mechanisms for the regulation of extracellular matrix organization, which require further study.

## Strength And Limitation

In this study, LC-MS/MS DIA and PRM technologies were applied to the human myometrium for the first time, and combined with transcriptomics analysis, it provided evidence for discovering molecular and functional changes of the myometrium during labor at the mRNA and protein level. More transcripts were identified than proteins; one reason for this difference could be due to the higher sensitivities of RNA sequencing technologies, especially when compared to the limited dynamic range of current MS (41). In addition, cellular mRNA and protein abundance are not only affected by transcription and translation rate but also determined by mRNA stability, mRNA splicing, and protein degradation (42). Due to these issues, integrated proteotranscriptomic analysis can obtain more accurate gene expression information.

It is also important to note that the transcriptome and proteome data for this study were derived from total RNA and total protein of the entire tissue samples, which included not only uterine smooth muscle cells but also epithelial cells, fibroblasts, endothelial cells, and multiple types of immune cells. Consequently, the proteotranscriptomics changes observed in this study were not confined to those occurring only in uterine smooth muscle cells. For a more complete picture of the labor process, single-cell omics may need to be used in investigating myometrial cell-specific changes and determining the dynamics of various cell types and proportions in quiescent and active laboring state.

## Conclusion

The results of our thoroughly analyzed proteotranscriptomics data, which describe alterations of mRNA and protein between NL and IL myometrium, showed a global increase in molecular functions primarily regulating inflammation under hypoxia stress during labor. This study provides good evidence at the transcriptome and proteome levels and therefore confirming the existing, and discovering new, molecular and biological changes during labor. Inflammation- and hypoxia-related pathway and mRNAs/proteins in our findings may be the targets for uterine contraction regulation, which is promising for abnormal labor management.

## Data Availability Statement

The datasets presented in this study can be found in online repositories. The names of the repositories and accession number(s) can be found in the section “*Materials and Methods*”.

## Ethics Statement

The studies involving human participants were reviewed and approved by the ethics committee of Guangzhou Women and Children Medical Center (No. 201915401). The patients/participants provided their written informed consent to participate in this study. Written informed consent was obtained from the individual(s) for the publication of any potentially identifiable images or data included in this article.

## Author Contributions

HL designed the research. LC and KJ analyzed the data and wrote the paper. YL, LW, QH, and JB performed the research. All authors contributed to manuscript revision, read, and approved the submitted version.

## Funding

This study was supported by the National Natural Science Foundation of China (81871181), the Foundation of Guangzhou Municipal Science and Technology Bureau (20210201040113), and the High-tech Major Featured Technology Program of Guangzhou Municipal Health Commission (2019GX07).

## Conflict of Interest

The authors declare that the research was conducted in the absence of any commercial or financial relationships that could be construed as a potential conflict of interest.

## Publisher’s Note

All claims expressed in this article are solely those of the authors and do not necessarily represent those of their affiliated organizations, or those of the publisher, the editors and the reviewers. Any product that may be evaluated in this article, or claim that may be made by its manufacturer, is not guaranteed or endorsed by the publisher.

## Abbreviations

RNA-seq, RNA sequencing; LC-MS/MS, quantitative liquid chromatography–tandem mass spectrometry; DIA, data-independent acquisition; PRM, parallel reaction monitoring; DDA, data-dependent acquisition; HCD, higher energy collision dissociation; 2-DE, two-dimensional gel electrophoresis; qPCR, real-time quantitative PCR; FPKM, kilobase of exon per million fragments mapped; FASP, filter-aided sample preparation; FDR, false discovery rate; DE, differential expressed; GO, gene ontology; BP, biological processes; CC, cellular components; MF, molecular functions; PCA, principal component analysis; GSEA, gene set enrichment analysis; PPI, protein–protein interaction.
